# Déjà vu experiences in healthy subjects are unrelated to laboratory tests of recollection and familiarity for word stimuli

**DOI:** 10.3389/fpsyg.2013.00881

**Published:** 2013-11-27

**Authors:** Akira R. O’Connor, Chris J. A. Moulin

**Affiliations:** ^1^School of Psychology and Neuroscience, University of St AndrewsSt Andrews, UK; ^2^Laboratoire d’Etude de l’Apprentissage et du Développement (CNRS UMR 5022), Université de BourgogneDijon, France

**Keywords:** déjà vu, memory, recognition, recollection, familiarity

## Abstract

Recent neuropsychological and neuroscientific research suggests that people who experience more déjà vu display characteristic patterns in normal recognition memory. We conducted a large individual differences study (*n* = 206) to test these predictions using recollection and familiarity parameters recovered from a standard memory task. Participants reported déjà vu frequency and a number of its correlates, and completed a recognition memory task analogous to a Remember-Know procedure. The individual difference measures replicated an established correlation between déjà vu frequency and frequency of travel, and recognition performance showed well-established word frequency and accuracy effects. Contrary to predictions, no relationships were found between déjà vu frequency and recollection or familiarity memory parameters from the recognition test. We suggest that déjà vu in the healthy population reflects a mismatch between errant memory signaling and memory monitoring processes not easily characterized by standard recognition memory task performance.

## INTRODUCTION

Theories of the déjà vu phenomenon derive from two distinct literatures: individual differences research and cognitive/neuroscientific investigations of familiarity processes (see Brown, 2003; O’Connor and Moulin, 2010 for reviews). The cognitive and neuroscientific literatures point to there being a disruption or misinterpretation of the *familiarity* signal that underlies recognition. One prominent idea is that there is an overlap between a perceptual experience (which is responsible for triggering the déjà vu) and a previously stored representation. The experient is unaware of the source of this evoked familiarity, leading to a feeling of déjà vu. This account lends itself to laboratory experimentation with the recognition without identification paradigm, where it is possible to make a stimulus familiar in such a way that the participant is not aware of the source of the familiarity (e.g., Cleary, 2008; Cleary and Reyes, 2009; Cleary et al., 2009, 2012). Such research places an emphasis on the role of familiarity in triggering déjà vu. In the laboratory, participants report feeling more déjà vu for items which share some overlap with a previously experienced stimulus, but only when this familiarity is not pinpointed to a previous experience.

More recent developments in the déjà vu literature have seen researchers draw on the dual-process theory of recognition memory. Dual-process theory differentiates familiarity from a second, less ambiguous recognition process, *recollection* (e.g., see Yonelinas, 2002; Diana et al., 2006 for reviews). Empirical studies that differentiate recollection from familiarity have typically found that recollection is slower, more accurate and associated with higher subjective confidence than familiarity. Recollection can confirm that memoranda which elicit high familiarity are “old” by providing an unambiguous contextual memory from the moment of encoding. It can reduce susceptibility to memory distortions such as misattribution (i.e., the act of attributing a partially retrieved memory to an incorrect source). And crucially, it is proposed that one function of recollection is as a self-monitoring strategy to minimize memory errors by disambiguating potentially unreliable familiarity signals. The use of strategic memory monitoring using recollection thereby avoids “illusions of familiarity” (Mandler, 1980; Jacoby et al., 1989; Whittlesea, 1993) in a process referred to as “recollection rejection” (Brainerd et al., 2003).

A déjà vu hypothesis which relies on the recollection rejection mechanism posits that in order to experience déjà vu one needs intact recollection to produce the clash in evaluations (see Spatt, 2002 for a neurological account of this view). That is, an inappropriate feeling of recognition will be generated, driven by an erroneous feeling of familiarity. But, in order for this to be experienced as déjà vu, it is critical that a separate memory monitoring process gives rise to the belief, knowledge or feeling that this is false. This view of déjà vu receives support from the temporal lobe epilepsy literature. Martin et al. (2012) took two groups of temporal lobe epilepsy (TLE) patients, some of whom did and some of whom did not have déjà vu as part of their seizure manifestation. They show that the patients with TLE who experience déjà vu have a familiarity disorder, as we would predict here. However, a disorder in familiarity did not differentiate those who did and did not experience déjà vu – both their groups of TLE patients were less able than controls to use familiarity signals to judge whether or not a stimulus had been previously seen. It was recollection that differentiated the groups – TLE patients who had déjà vu were *better* at using recollection to tell targets from lures than TLE patients without déjà vu. The interpretation of this finding is that recollection is required to be aware of the erroneous familiarity at play in the déjà vu phenomenon. Patients with TLE who have both familiarity and recollection deficits do not possess sufficient memory capacity to detect the error of familiarity. In this experiment we take these findings and a similar task (Experiment 1 from Martin et al., 2012) to a group of healthy undergraduates to see if we can find a similar relationship between recollection and déjà vu experiences.

The individual difference literature is reviewed in Brown (2003) and comprehensively detailed in Brown (2004). As an example, associations are found with the degree to which people travel. Chapman and Mensh (1951) found that only 11% of those who did not travel experienced déjà vu, with the number increasing to 32% for those who traveled up to five times a year. Richardson and Winokur (1967) also found increased reports of déjà vu when they compared those who traveled with those who did not. Associations between the propensity for the experience and other individual differences have been used to investigate the triggers of and bases for déjà vu. For instance, given a null predictive effect of déjà vu on dissociative experiences in a sample of 227 healthy participants, Adachi et al. (2008) concluded that déjà vu was not a dissociative experience. Whilst the precise mechanism by which travel frequency provides increased opportunity for déjà vu experiences remains open, the robust association and the inter-individual variability in travel frequency make it an ideal variable to use as an individual difference association check in déjà vu assessments on otherwise homogenous (e.g., age-restricted) samples.

In a neat integration of the individual differences and neuroscientific literatures, Brázdil et al. (2012) combined questionnaire and imaging methods to search for structural correlates of déjà vu. In 113 healthy participants who had been administered the Inventory for Déjà vu Experiences Assessment (IDEA; Sno et al., 1994), they also measured grey matter volume in brain structures critical for recognition. Using an initial split of the sample according to whether or not they had ever experienced déjà vu, they identified brain regions whose morphology differed according to déjà vu experience. Grey matter volume from these regions was then correlated with frequency estimates from the questionnaire, with the finding that it was negatively associated with déjà vu experiences, particularly in mesial temporal/hippocampal regions. That is, people who experienced déjà vu had lower grey matter volume of areas critical for recognition memory (though no differentiation between neural correlates of familiarity and recollection was made). Notably, in Brázdil’s study there were no behavioral measures of memory processes which, using a similar design and a larger sample, we attempt here. Given that we can observe differences in temporal lobe regions according to the frequency of déjà vu experiences, we might expect that there is a behavioral corollary.

The neuroscientific work of Martin and Brázdil leads us to predict that familiarity and recollection should be related to déjà vu experiences in a group of healthy students, and generates testable hypotheses about recognition memory performance and déjà vu. These predictions differ according to which prior study is in question. Brázdil’s work on the temporal lobe suggests a reduction in volume for the people who experience déjà vu. Thus we might expect a diminution of performance in recognition memory associated with increased déjà vu experiences. Brázdil et al. (2012) do not indicate whether they think that familiarity or recollection is the process at play in déjà vu. According to Martin et al. (2012) we expected that people who have déjà vu should have high recollection scores, in line with Martin et al.’s behavioral results in TLE. In contrast, the work of Cleary and colleagues points to an opposing prediction: because it is detected familiarity for a past event which is not recalled, we might expect that déjà vu is caused by a lack of recollection (e.g., Cleary, 2008; Cleary and Reyes, 2009; Cleary et al., 2009, 2012). In the current study we tested these hypotheses with an individual differences approach. Participants took part in a standard recognition memory paradigm which asked for evaluations of recollection and familiarity via a Remember-Know procedure, and we compared these scores with the self-reported incidence of déjà vu, and a variable known to be correlated with incidence: travel frequency.

## MATERIALS AND METHODS

### PARTICIPANTS

Participants were 206 University of Leeds undergraduates (173 female) who completed the experiment as part of a laboratory class. Informed consent was obtained in accordance with the Institute of Psychological Sciences Ethics Committee at the University of Leeds.

### STIMULI

A set of 80 words, chosen from the Gilhooly and Logie (1980) 1944-word database, was used for all participants. There were 40 high frequency words (min. familiarity rating 6.20; e.g., *room*) and 40 low frequency words (maximum familiarity rating 2.40; e.g., *aperture*).

### DESIGN AND PROCEDURE

The experiment lasted approximately 15 min and was administered using Superlab Pro (version 2.0.2, Cedrus Software) on IBM-compatible PCs. Participants were given the on-screen instructions “Try and memorize each word for a later test,” before beginning the study phase consisting of 40 study trials. During study, they were presented with 20 high frequency words and 20 low frequency words in a randomized order. Each study trial word was presented in black 48 pt Tahoma font on a white background for 1,000 ms. A blank white screen lasting 500 ms followed each study trial.

After the final study trial, the test response options were explained onscreen and participants were given the opportunity to familiarize themselves with the terminology they would use at test. The definitions provided to participants were as follows:

*Remember* - If you can remember contextual information regarding your own thoughts as you initially saw the word e.g., “I thought of a striped horse” [zebra], press the 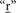 key.

*Familiar* - If you cannot remember anything contextual but are familiar with the word from recent exposure to it, press the 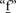 key.

*Guess* - If you simply think you saw the word before but cannot be sure, press the 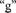 key.

*New* - If you believe that the word is a new word not previously presented to you, press the 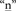 key.

Once participants were happy to proceed, they began the test phase. The test comprised 80 words (40 previously presented targets, 20 high frequency lures and 20 low frequency lures) presented in a randomized order. Each test trial word was presented in black 48 pt Tahoma font on a white background for 10,000 ms or until a keyboard response with made. A blank white screen lasting 500 ms followed each test trial.

After completing the test phase, participants responded to a series of postexperimental questions including those assessing gender, age^1^, déjà vu incidence, jamais vu incidence and travel frequency as follows:

*Déjà vu incidence*: In the past 6 months, how many times have you had the feeling of having experienced a sensation or situation before in exactly the same way, when in fact you were experiencing it for the first time?

*Jamais vu incidence:* In the past 6 months, how many times have you had the feeling that you had never experienced a sensation of situation before when in fact you had (the opposite of déjà vu)?

*Travel frequency*: Approximately how many times in the past 6 months have you traveled more than 100 km from your home?

For the three questions above, participants responded using the number keys zero to six to indicate that exact value, or seven to indicate “seven or more”.

## RESULTS

### RECOGNITION PERFORMANCE

Recognition memory accuracy was high, as evidenced by the hit and correct rejection rates. The mean overall hit rate (proportion “old” responses to targets; includes “Remember,” “Familiar” and “Guess” responses) was 0.819 (SD = 0.119) and the mean correct rejection rate (proportion “New” responses to lures) was 0.742 (SD = 0.202). A frequency-based mirror effect (Glanzer and Adams, 1990) was observed in the proportions of hits and correct rejections to high and low frequency words. The mean proportion of hits to high frequency targets (0.757, SD = 0.167) was significantly lower than to low frequency targets (0.879, SD = 0.115), *t*(205) = 11.69, *p* < 0.001, *d* = 2.38. Complementing this difference, the mean proportion of correct rejections to high frequency lures (0.690, SD *=* 0*.*249) was also significantly lower than to low frequency lures (0.792, SD = 0.185), *t*(205) = 8.41, *p* < 0.001, *d* = 0.62.

Sensitivity (*d′*) and bias (*c*) parameters were calculated from the hit and correct rejection rates above using formulae from the equal variance signal detection model (Green and Swets, 1966; Macmillan and Creelman, 2005), and corrected for errorless responding (Snodgrass and Corwin, 1988). The mean overall *d′* was 1.63 (SD = 0.65) whilst *c* was **-**0.12 (SD = 0.43). When parameters for high and low frequency words were compared, in keeping with the frequency-based mirror effect, mean *d′* for low frequency words (2.11, SD = 0.77) was significantly greater than mean *d′* for high frequency words (1.32, SD = 0.76), *t*(205) = 16.50, *p* < 0.001, *d* = -1.15. Despite the sensitivity difference, there was no significant difference in *c* for low and high frequency words (-0.16, SD = 0.43 and -0.10, SD = 0.58 respectively), *t*(205) = 16.50, *p* = 0.053, *d* = 0.14.

### RECOGNITION RESPONSES

The proportions of recognition responses to targets are summarized in **Table 1**. Crucially for subsequent analyses, the proportion of “Remember” responses is well below ceiling, and affords the potential for interindividual variability in the recollection parameters reported in Section “Recollection and Questionnaire Responses” onwards.

**Table 1:** Proportion recognition responses to targets.

	“Remember”	“Familiar”	“Guess”
High Frequency	0.467 (0.238)	0.164 (0.149)	0.126 (0.122)
Low Frequency	0.610 (0.226)	0.188 (0.181)	0.084 (0.091)

*Mean (standard deviation in parentheses) proportions of all targets responded to within the allotted 10,000 ms per trial.*

**FIGURE 1:**
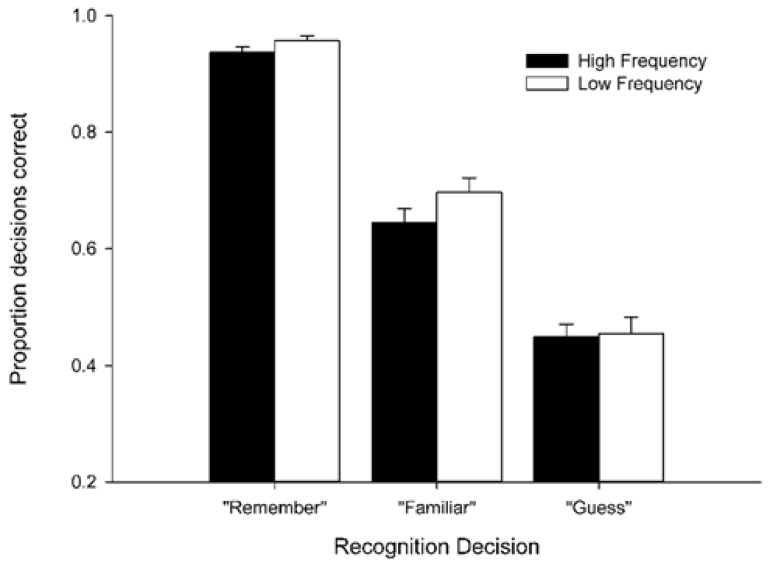
**Proportion targets comprising each recognition response category.** Bars show mean decision accuracy for all responses made within the allotted 10,000 ms per trial. Error bars represent standard error of the mean.

To check that participants were using “Remember” and “Familiar” responses in a manner consistent with their intended use, we compared the proportions of targets that made up each response subcategory (see **Figure 1**). The proportions from 141 participants for whom it was possible to calculate these values were entered into a 2 (word frequency) × 3 (response type) repeated measures factorial ANOVA. (It was not possible to calculate these proportions for participants who did not provide a single instance of particular response e.g., “Guess” responses to high frequency words, so these participants were excluded from the analyses reported here.) There was a trend towards a main effect of word frequency, with recognition responses to low frequency words numerically more likely to be given to targets (0.703, SD = 0*.*331) than recognition responses to high frequency words (0.677, SD = 0*.*305), *F*(1,140) = 3.84, *p* = 0.052, $\eta_{p}^{2}$ = 0.027. Importantly, there was a significant main effect of response type, *F*(2,280) = 253.86, *p* < 0.001, $\eta_{p}^{2}$ = 0.645. Bonferroni-corrected pairwise comparisons showed that “Remember” responses comprised a significantly greater proportion of targets (0.947, SD = 0.105) than “Familiar” responses (0.671, SD = 0.284) and “Guess” responses (0.453, SD = 0.299), and “Familiar” responses in turn comprised a significantly greater proportion of targets than “Guess” responses, all *p*s < 0.001. There was no significant ANOVA interaction, *F* < 1. This pattern of results is entirely consistent with the participants being aware of the phenomenological differences between “Remember,” “Familiar” and “Guess” responses, and using them appropriately. These results provide a validation of the descriptives reported in **Table 1** as appropriate measures of recollection and familiarity to be explored in subsequent analyses.

### QUESTIONNAIRE RESPONSES

Mean responses to postexperimental questionnaire items and their inter-item correlations are summarized in **Table 2**. Of particular interest are déjà vu experiences, which were reported significantly more than jamais vu experiences, *t*(204) = 11.14, *p* < 0.001, *d* = 0.78. The differences in reported incidence are also reflected in the proportions of participants reporting no occurrence of each experience in the previous 6 months, with only 0.16 of the sample reporting no déjà vu, compared to 0.66 making the equivalent judgment of jamais vu occurrence.

**Table 2:** Postexperimental questionnaire descriptive statistics and correlations.

		Déjà vu incidence	Jamais vu incidence	Travel frequency
Descriptives	Mean (SD)	2.74 (2.11)	0.82 (1.59)	4.81 (2.26)
Correlations*r* (*n*, *p*)	Déjà vu incidence		0.129 (205, 0.066)	0.216^**^ (205, 0.002)
	Jamais vu incidence			0.104 (205, 0.139)

** Significance at the 0.01 level. One participant did not provide ratings for jamais vu incidence and travel frequency, which is reflected in the modified ns listed for correlations involving these ratings. Means are likely to be underestimations of actual occurrence as the response option 7 represents “7 or more” occurrences of the experience in the last 6 months.

We also observed relationships between variables in the postexperimental questionnaire predicted from previous individual differences research. There was a significant positive correlation between déjà vu incidence and travel frequency, *r*(203) = 0.216, *p* = 0.002. Also of note is the trend towards a significant positive correlation between the self-reported incidence of déjà vu and jamais vu, *r*(203) = 0.129, *p* = 0.066. This may reflect covarying tendencies to either experience these subjective memory sensations, or to report them.

#### Recollection and questionnaire responses

Recollection parameters were calculated by subtracting the proportion of “Remember” responses to lures from the proportion of “Remember” responses to targets. This was conducted for both high and low frequency words, yielding two parameters, *Rec*_high_ and *Rec*_low_. The equivalent calculation for “Familiar” responses yielded *Fam*_high_ and *Fam*_low_ parameters. Mean *Rec* and *Fam* parameters, their inter-parameter correlations and correlations with the individual difference measures of travel frequency and overall *d′* (collapsed across high and low frequency words), are summarized in **Table 3**. (Both *Rec*_high_ and *Fam*_high_ parameters were significantly larger than their low frequency equivalent parameters, *t*(205) = 10.09, *p* < 0.001 and *t*(205) = 2.05, *p* = 0.042, respectively, and so they have been examined as separate parameters in these analyses.) These correlations go some way towards acting as a manipulation check to establish the validity of the *Rec* and *Fam* parameters.

**Table 3:** Recognition response descriptive statistics and correlations.

		*Rec*_high_	*Rec*_low_	*Fam*_high_	*Fam*_low_
Descriptives	Mean (SD)	0.425 (0.239)	0.580 (0.226)	0.049 (0.186)	0.110 (0.214)
Correlations *r* (*n*, *p*)	Travel frequency	0.006 (205, 0.930)	-0.040 (205, 0.572)	0.062 (205, 0.379)	-0.028 (205, 0.686)
	*d′*	0.627^***^ (206, <0.001)	0.340^***^ (206, <0.001)	0.261^***^ (206, <0.001)	0.272^***^ (206, <0.001)
	*Rec*_high_		0.638^***^ (206, <0.001)	-0.353^***^ (206, <0.001)	-0.258^***^ (206, <0.001)
	*Rec*_low_			-0.422^***^ (206, <0.001)	-0.654^***^ (206, <0.001)
	*Fam*_high_				0.593^***^ (206, <0.001)

Rec and Fam represent recollection and familiarity parameters, respectively, with subscript high and low indicating the frequency of stimuli for which the parameters are calculated. d′ represents the sensitivity parameter collapsed across both word frequency conditions; ^***^denotes significance at the 0.001 level. One participant did not provide ratings for travel frequency, which is reflected in the modified ns listed for correlations involving this rating.

As expected, there were no correlations between *Rec* and *Fam* parameters and travel frequency (all *p*s > 0.379). There were significant correlations between both *Rec*_high_ and *Rec*_low_ and *d′*, which unsurprisingly indicates that selective use of recollection to disambiguate targets from lures is associated with greater overall performance on the memory task, *r*(204) = 0.627, *p* < 0.001 and *r*(204) = 0.340, *p* < 0.001, respectively. Significant positive correlations were also found between both *Fam*_high_ and *Fam*_low_ and *d′*, *r*(204) = 0.261, *p* < 0.001 and *r*(204) = 0.272, *p* < 0.001, respectively. Taken together, these correlations simply demonstrate the positive contributions to *d′* of *Rec* and *Fam* responses to targets, combined with the negative contributions to *d′* of the same responses to lures. Combined with the cross-frequency correlations, in which recollection parameters for each word frequency category were positively correlated with the other frequency category recollection parameter, and negatively correlated with both familiarity parameters (all *p*s < 0.001), these relationships suggests that participants were utilizing recollection and familiarity in a stable and reliable manner to disambiguate targets from lures.

### RECOLLECTION AND DÉJÀ VU

Three critical analyses were conducted on the relationships between recollection and familiarity parameters and individual differences in déjà vu experience. In the first, we conducted bivariate correlation analyses of the *Rec* and *Fam* parameters and self-reported déjà vu incidence from the postexperimental questionnaires (section Simple Correlations). In the second, we restricted our sample to those with unusually low *Fam* parameters, analogous to impaired familiarity, and repeated the correlation analyses to establish whether a more nuanced relationship between *Rec* parameters and déjà vu exists in those with recognition performance akin to impaired familiarity-based discrimination (section Restricted Sample Correlations). In the third, we conducted logistic regression analyses using the *Rec* and *Fam* parameters as predictors, with the aim of classifying those who had and had not experienced déjà vu in the past 6 months (section Logistic Regression). Across all three analyses, we found no relationship between recollection and familiarity parameters and self-reported déjà vu incidence.

#### Simple correlations

**FIGURE 2:**
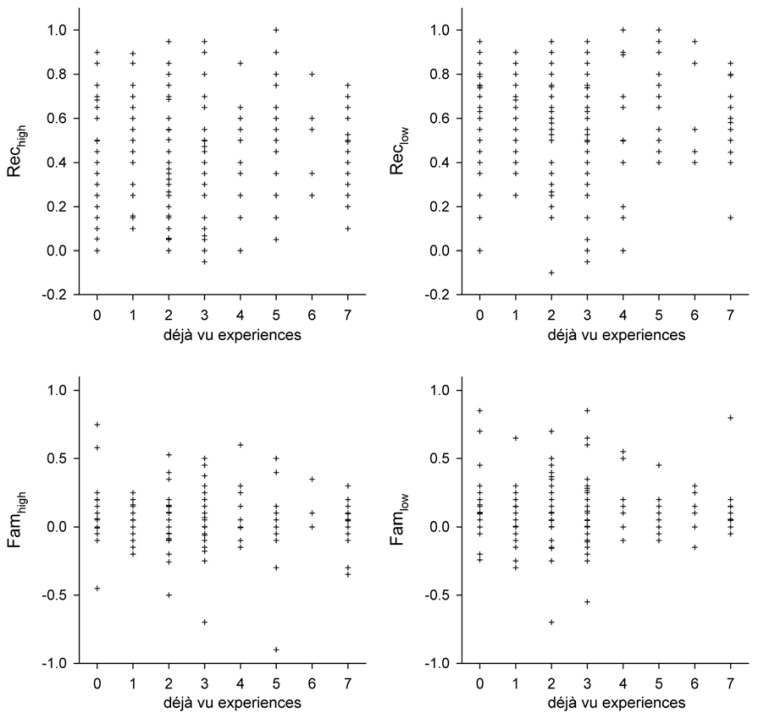
**Déjà vu-*Rec* and Déjà vu-*Fam* scatterplots.** For all plots, reported déjà vu incidence is plotted on the *x*-axis. *y*-axis plot *Rec* parameters (top panels; calculated as “Remember” responses to targets minus “Remember” responses to lures) and *Fam* parameters (bottom panels; calculated as “Familiar” responses to targets minus “Familiar” responses to lures). Separate *Rec* and *Fam* parameters were calculated for responses to high frequency (left panels) and low frequency (right panels) words.

Scatterplots illustrating the simple correlation data are shown in **Figure 2**. There were no significant correlations between *Rec*_high_ and déjà vu incidence, *r*(204) = 0.042, *p* = 0.552, or *Rec*_low_ and déjà vu incidence, *r*(204) = 0.029, *p* = 0.683. In equivalent analyses of familiarity responses, *Fam*_high_ and *Fam*_low_ were not found to be significantly correlated with déjà vu incidence, *r*(204) = -0.026, *p* = 0.716 and *r*(204) = -0.032, *p* = 0.649, respectively. Overall, there was no simple association between the tendency to differentially employ either recollection or familiarity and the tendency to experience déjà vu.

#### Restricted sample correlations

The tested hypotheses posit the cause of déjà vu as either erroneous familiarity that is disconfirmed or erroneous familiarity that is left unchecked – déjà vu experients should feel familiarity for a novel episode, but it is the interaction with one or other extreme of recollection processing that drives the experience. The analog of this amongst our sample would be people who had poor discrimination in terms of familiarity and either excellent recollective discrimination or poor recollective discrimination being those who experience déjà vu the most. In the first restricted sample correlation analyses, we selected a *Fam*_high_ threshold of -0.05, above which participants were excluded from the analysis. This left 34 participants in the subsample whose “Familiar” judgements are characterized by particularly poor target-lure discrimination. As was expected given the selection criterion, the mean *Fam*_high_ parameter from the subsample (-0.208, SD = 0.184) was significantly lower than the population mean (0.049) inferred from the complete sample, *t*(33) = 8.14, *p* < 0.001. Conversely, there was no significant difference in the subsample’s mean *Rec*_high_ parameter (0.445, SD = 0.243) and the population mean (0.425), *t*(33) = 0.488, *p* = 0.629. This suggests that within the subsample of 34 participants whose “Familiar” judgements do not discriminate targets from lures, the overall “Remember” response discrimination is representative of the full sample. Even within this subsample, the resulting correlation between *Rec*_high_ and déjà vu showed no significant relationship, *r*(32) = -0.051, *p =* 0.774.

A second restricted sample correlation analysis echoed the first, this time using parameters from the low frequency words. We selected a *Fam*_low_ threshold of -0.04, yielding 35 participants whose mean *Fam*_low_ parameter (-0.157, SD = 0.139) was significantly lower than that of the population mean (0.110), *t*(34) = 11.15, *p* < 0.001. This time, the subsample’s *Rec*_low_ parameter (0.711, SD = 0.185) was significantly higher than the population mean (0.580), *t*(34) = 4.20, *p* < 0.001. The resulting correlation between *Rec*_low_ and déjà vu once again showed no significant relationship between the two measures, within the subsample, *r*(33) = 0.097, *p =* 0*.*579. Across both restricted sample correlation analyses conducted on respondents whose “Familiar” responses do not discriminate targets from lures, we found no evidence that accurate recollection is associated with déjà vu incidence.

#### Logistic regression

Finally, two logistic regression analyses were conducted to predict the report of one or more déjà vu experience in the previous 6 months using the whole dataset. These analyses aimed to establish whether the frequency of déjà vu experience (dichotomised as those who experience déjà vu frequently, i.e., at least one episode within the past 6 months, versus those who experience it infrequently i.e., no episode within the past 6 months) can be predicted by the recollective experience parameters recovered using the memory test. The first used *Rec*_high_ and *Fam*_high_ as predictors of déjà vu. When tested against the constant-only model, the experimental model was statistically nonsignificant, indicating that neither predictor reliably differentiated those reporting at least one déjà vu experience from those reporting none, χ^2^(2) = 0.758, *p =* 0*.*685. Nagelkerke’s *R*^2^ of 0.006 indicated a negligible relationship between prediction and grouping, with prediction success rate of 84%, but crucially, 0% accuracy in predicting those who had not experienced déjà vu.

The second logistic regression model used *Rec*_low_ and *Fam*_low_ as predictors, but again failed to improve upon the constant-only model, χ^2^(2) = 4.55, *p =* 0*.*103. Nagelkerke’s *R*^2^ of 0.037 once again indicated a negligible relationship between prediction and grouping and, with identical prediction rates to the previous model, also failed to predict any of those who had not experienced déjà vu. Across both logistic regression models, and all analyses of association reported, there was no significant relationship between individual differences in recognition memory parameters and déjà vu incidence.

## DISCUSSION

Recent studies have proposed that there is a relationship between recognition memory (especially as subdivided into recollection and familiarity and studied in the context of the temporal lobe) and déjà vu experiences. Since déjà vu experiences are difficult to produce in the laboratory, a scientific line of enquiry has involved using individual differences to explore the factors which influence the frequency of the experience. We used this individual differences approach on healthy subjects to examine how recognition memory test scores relate to the experience.

Our recognition memory test replicated standard word frequency effects, with performance reliably above chance. With our individual difference measures we replicated the typical correlation between déjà vu frequency and frequency of travel. In short, we have a well-powered memory experiment and a correlational sample which replicates the established association with travel frequency from the déjà vu literature, but which points to there being no relationship between the tendency to experience déjà vu and memory performance or recollective experience reported during the test.

We expected to find a relationship between memory measures and the frequency of déjà vu experience. Within a sample of TLE patients, Martin et al. (2012) found that recognition test scores contrasting recollection and familiarity differentiate those people who do and do not experience déjà vu. Brázdil et al. (2012) found that in healthy participants, there are differences in the brain structures of those people who do or do not experience déjà vu. These findings raise the possibility of observing memory test score differences in the healthy population according to the frequency of déjà vu experiences. That we found no relationship between déjà vu and recollection – especially in such a large sample and where other correlations with the déjà vu experience were observed – points to the fact that the experience is not captured solely in terms of differences in the capacity for people to experience recollection or familiarity in a test of recognition memory. Nevertheless, given the limitations associated with this interpretation of a null effect, we conducted a sensitivity power analysis to determine the minimum correlation coefficient we could reliably detect with our sample. Based on a two-tailed alpha value of 0.05, a power (1 – beta) value of 0.95 and our sample size of 206, we recovered a minimum detectable correlation coefficient of 0.247. To reliably detect a smaller correlation coefficient would require a larger sample size than that which we tested. Put another way, even if there were a correlation of just below *r* = 0.247, the variance in déjà vu incidence accounted for by parameters recovered from standard tests of recognition would not exceed 6.1%. Of course, it remains possible that measurable correlations between recollection processes and déjà vu could be observed in other special groups. Nonetheless our data suggest that there is not a relationship between recollection and déjà vu in the neurologically normal population.

If we assume that these data represent the true absence of an association between recollection and déjà vu, how should they inform cognitive theories of the déjà vu phenomenon? Elsewhere, we have suggested that déjà vu is a random neurological event (e.g., O’Connor and Moulin, 2010), echoing the claim of Penfield (1955) that déjà vu was akin to a subclinical minor epileptic event. This means that although memory systems are implicated in the déjà vu experience, it might not be individual differences in these systems which capture differences in the frequency with which people have the experience. Instead, other individual difference variables show a relationship with déjà vu, presumably which influence the readiness to experience the event or the ability to articulate it. In fact, we find that although a test of recognition memory does not correlate with déjà vu, a non-cognitive variable, travel frequency, does. To explain this result we have previously applied an attribution discrepancy argument (Whittlesea and Williams, 1998). That is, one will be more likely to detect déjà vu in situations where the mismatch between the feeling of familiarity and the environment is more apparent. Thus, the more one travels to novel places, the more opportunities there are to experience déjà vu, assuming that it is less likely to occur in mundane contexts. To experience déjà vu, one has to detect that the feeling of familiarity is in fact false. A novel context should aid this detection process.

Taking our null findings to indicate the absence of a relationship between memory measures and the tendency towards déjà vu experience is inconsistent with the findings from the TLE sample of Martin et al. (2012). However, it should be stressed that their neuropsychological sample represents a set of patients with clear temporal lobe pathology compared to our sample of healthy undergraduates. Furthermore, déjà vu is often experienced as “meaningful” or distressing in the TLE population, as well as being reported more frequently (see Illman et al., 2012 for a review of déjà vu in TLE). Thus, it seems reasonable that the TLE déjà vu is a product of the unusual morphology or damage to the temporal lobe. The fact that differences in déjà vu experiences are reflected in recognition memory test scores is possibly just because the temporal lobe is implicated in both. Damage to different structures of the temporal lobe is reflected in the scores on tests of recollection and familiarity, but this merely indicates the structures at play in the experience, rather than implicating the process of recollection in the déjà vu experience. In short, TLE déjà vu may be caused by differential pathology of the temporal lobe which is revealed in tests using recollection and familiarity, but in the intact temporal lobe, tests of recollection and familiarity do not capture the nuances of the experience.

Another methodological factor within the present study which could conceivably account for the lack of an association between basic memory processes and déjà vu the nature of stimuli used in our recognition test. Other groups investigating déjà vu have typically used rich visual stimuli reasoning that déjà vu is usually encountered when faced with this sort of stimulus (e.g., Cleary and Reyes, 2009; Martin et al., 2012). We chose to use visually presented word stimuli because we were not trying to generate déjà vu but were attempting to show an association between established basic memory processes, which word stimuli are used to reliably recover (for a review see Yonelinas, 2002), and déjà vu incidence. It remains possible that differences between the memory processes applied to complex visual stimuli and visually presented word stimuli could lead to the absence of correlation between déjà vu and the basic memory parameters recovered in this experiment, whereas an alternate experiment using rich visual stimuli would demonstrate such an association. If this finding was demonstrated however, it would imply a dissociation between recollection processes used for words and rich visual stimuli which in itself would be a major issue (and a new finding) for dual-process theories of recognition.

The Brázdil et al. (2012) data gave us our clearest prediction that in healthy undergraduates we might see systematic differences in recognition memory according to the frequency of déjà vu experiences. Again, this argument rests on anatomical differences in the temporal lobe, rather than behavioral markers of the memory system. Crucially here, our behavioral work does not support the idea that the propensity to experience déjà vu is due to a marked change in the functional capacity of the temporal lobe. One further consideration is that in their regions of interest analysis, Brázdil did not examine many structures outside the temporal lobe. The temporal lobe is often referred to as part of a memory network taking in the frontal lobes and it has been speculatively proposed that it is the interplay between these two regions which generates in déjà vu (see O’Connor and Moulin, 2010). While this proposal has yet to be empirically validated, there exists the possibility that the prefrontal cortex and yet other brain regions might alternatively capture the differences between those who do and do not experience déjà vu.

Our concluding remark is related to the contribution of the frontal lobes. The frontal lobes are often implicated in the strategic regulation, monitoring and interpretation of temporal lobe signals (e.g., Moscovitch, 1992). This collection of functions can be construed as being “metacognitive.” Our proposal here is that it may be individual differences in metacognition which explain the frequency of déjà vu experiences, rather than memory function *per sé*. One of the ways of thinking about metacognition is to consider it as reflecting the relationship between our memory system, and our experience of it. In the déjà vu experience, one must consciously reflect on the signals being generated by the memory system, and we assume that this requires a rich network of brain regions and a complex interaction of cognitive processes. For this reason, some authors have described the déjà vu experience as metacognitive (e.g., Arango-Muñoz, 2010; Moulin and Souchay, 2013), in the same way that other authors have described the failure of word retrieval, the tip-of-the-tongue experience as metacognitive (e.g., Bacon et al., 2007). Déjà vu and the tip-of-the-tongue experience both signal information to the experient about the ongoing processes in cognition, what have been called “epistemic feelings” (Arango-Muñoz, 2010). If there is an individual difference variable which might explain the susceptibility to the déjà vu experience, it may be the relationship we have with our memory. To experience déjà vu, one needs to be able to reflect on the mismatch between current feelings generated by the memory system, and the contents of consciousness, and current experience. Perhaps to do this captures more than mere performance on a recognition memory test. Future research could consider the relationship between different types of metacognitive experience in an individual differences design, and perhaps begin to evaluate how people experience and interpret their memory system.

To conclude, the null correlations between recognition performance and déjà vu experiences point to déjà vu remaining a difficult to research, infrequent and nebulous mental experience. It seems reasonable to continue to assume that it is essentially a quirk of the memory system, but déjà vu is not an experience which is captured in the general population by differences in the ability to recognize previously encountered information on the basis of recollection or familiarity.

## AUTHOR CONTRIBUTIONS

Akira R. O’Connor and Chris J. A. Moulin were jointly responsible for the design and conduct of the research, with testing taking place during a practical class led by Chris J. A. Moulin. Akira R. O’Connor and Chris J. A. Moulin collaboratively analysed the data and wrote the manuscript.

## Conflict of Interest Statement

The authors declare that the research was conducted in the absence of any commercial or financial relationships that could be construed as a potential conflict of interest.

## Footnotes

1 Within our undergraduate sample, 94% of participants indicated that they were in the 19–22 age range. Due to the highly limited variability in the age range of our sample, we do not examine the relationship between age and any other variables within the Results.
